# Tumor-to-tumor metastasis: A case report of metastasis of nasopharyngeal carcinoma to meningioma and review of the literature

**DOI:** 10.1097/MD.0000000000033500

**Published:** 2023-04-14

**Authors:** Xue Li, Min Jing, Yanbo Dai, Xiaoming Xing

**Affiliations:** a Department of Pathology, First People’s Hospital of Neijiang, Neijiang, China; b Department of Radiology, First People’s Hospital of Neijiang, Neijiang, China.

**Keywords:** meningioma, nasopharyngeal carcinoma, tumor-to-tumor metastasis

## Abstract

Tumor to tumor metastasis is a rare phenomenon clinically, especially tumor to meningioma metastasis. Here, we present an unusual case of metastasis of nasopharyngeal carcinoma to meningioma.

**Patient concerns:**

A 55-year-old man, with a history of nasopharyngeal carcinoma, developed neurological symptoms.

**Diagnosis:**

Computed tomography and magnetic resonance imaging revealed a mass on left temporoparietal lobe, indicating the presence of meningioma. The pathologist diagnosed the metastasis of nasopharyngeal carcinoma (differentiated non-keratinizing squamous cell carcinoma) to meningioma.

**Interventions:**

Chemotherapy and immunotherapy were performed following the resection.

**Outcomes:**

The patient has been well and no relapses has been observed.

**Lessons:**

Doctors should be aware of the presence of tumor-to-tumor metastasis, which is a rare phenomenon. A positive history of primary extracranial tumor should raise the suspicion of potential tumor-to-tumor metastasis.

## 1. Introduction

Metastasis from one tumor to another tumor, known as tumor-tumor metastasis (TTM),^[1]^ is a very rare phenomenon in clinical practice. There is one primary tumor as the recipient tumor and another tumor as the donor tumor in TTM. Meningioma, accounting for 36% in intracranial tumor,^[2]^ is the most common benign tumor to harbor tumor of metastasis.^[3]^ But only a few cases of tumor-to-meningioma have been reported. Here, we report a case of nasopharyngeal carcinoma metastasizing to meningioma.

## 2. Case report

**Figure 1.**
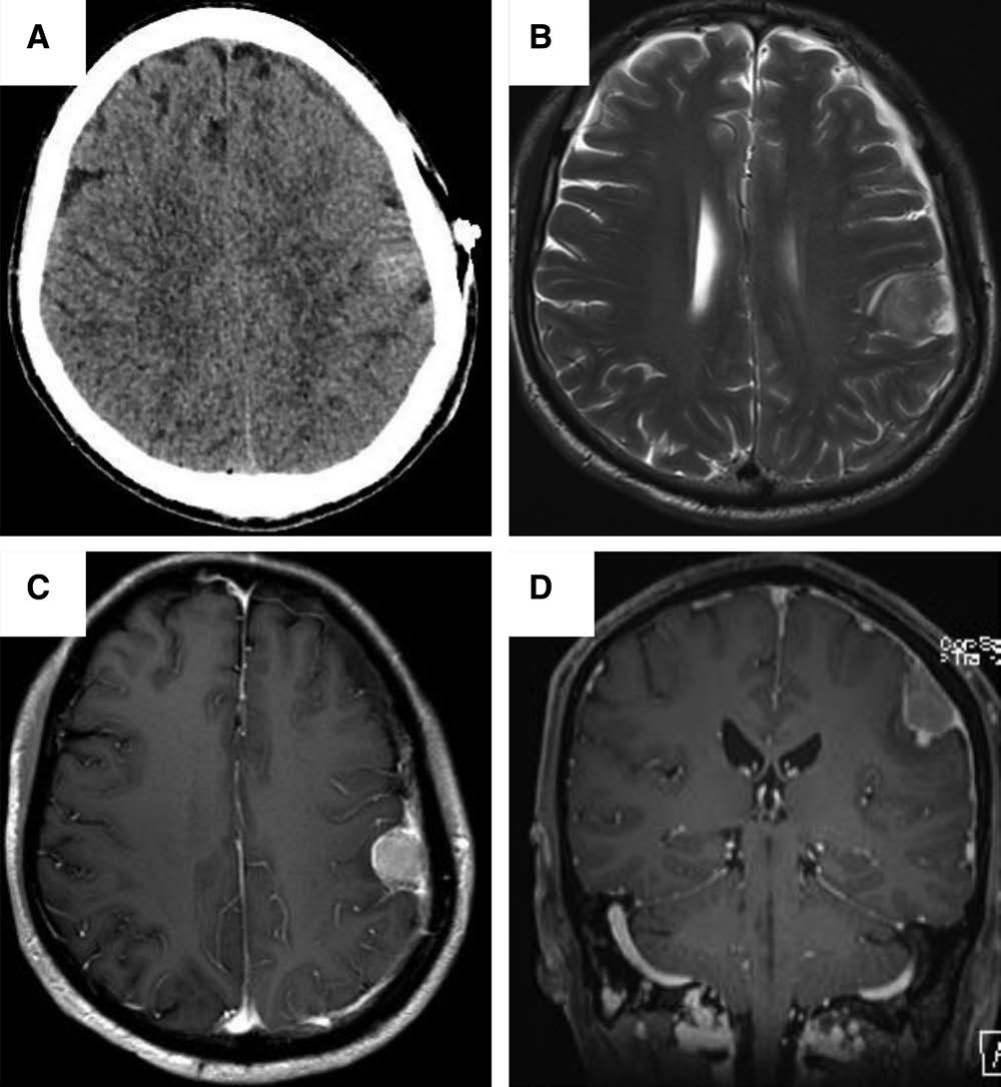
(A) (CT) A mass on the left temporoparietal lobe. (B) (MRI) Dural tail sign and adjacent meningeal thickening. (C and D) (enhanced MRI) Enhancement of the mass, small nodules on the margin. CT = computed tomography, MRI = magnetic resonance imaging.

In June 2022, a 55-year-old male presented with 2-hour history of numbness on the right side of face, weakness in the right upper extremity, and difficulty in speaking. Computed tomography and cranial magnetic resonance imaging revealed there was a well circumscribed mass of slightly high and uniformed density with a diameter of about 1.3 centimeter on the left temporoparietal lobe, with dural tail sign and adjacent meningeal thickening. There was obvious uniform enhancement and the edge of the mass was obviously enhanced with small nodules in enhanced magnetic resonance imaging (Fig. 1). There was no shift in the midline structure, and there was no abnormality in the ventricle, cerebellum brainstem or skull. Meningioma on left temporoparietal lobe was diagnosed before operation according to the imaging examination. The patient had a 1-year history of nasopharyngeal carcinoma with chemotherapy, immunotherapy and radiotherapy. Surgery was performed for the intracranial mass. Intraoperation found the tumor located on the junction of left temporoparietal lobe and the tumor base was located on the dura near the sagittal sinus. The tumor was gray-white in color, soft in texture, rich in blood supply with a diameter of about 2 cm. The tumor was encapsulated but adherent to adjacent brain tissue.

**Figure 2.**
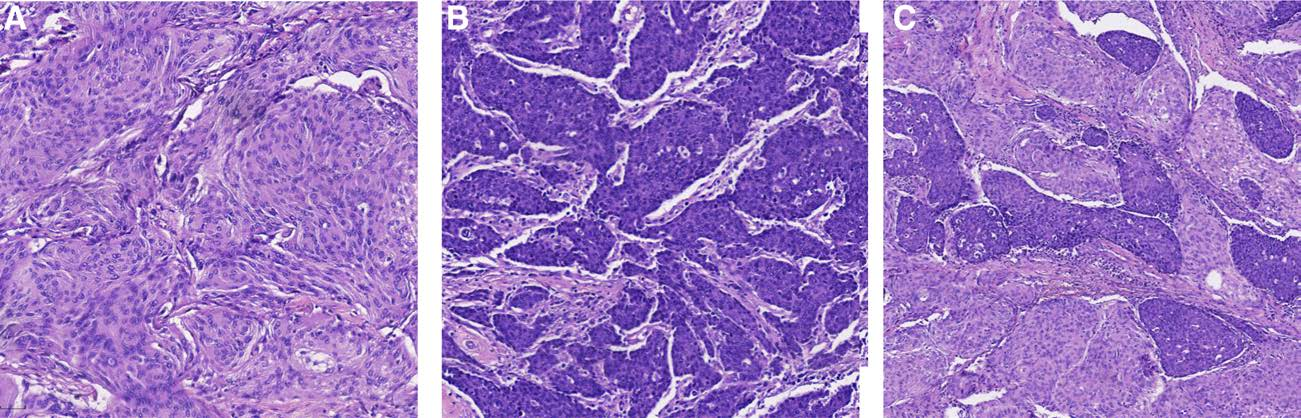
Histopathology of the mass. (A) Spindle cells arranged in parallel, fascicular and staggered. (B) Epithelioid cells in the form of nests and sheets. (C) Epithelioid cells were surrounded by spindle cells.

**Figure 3.**
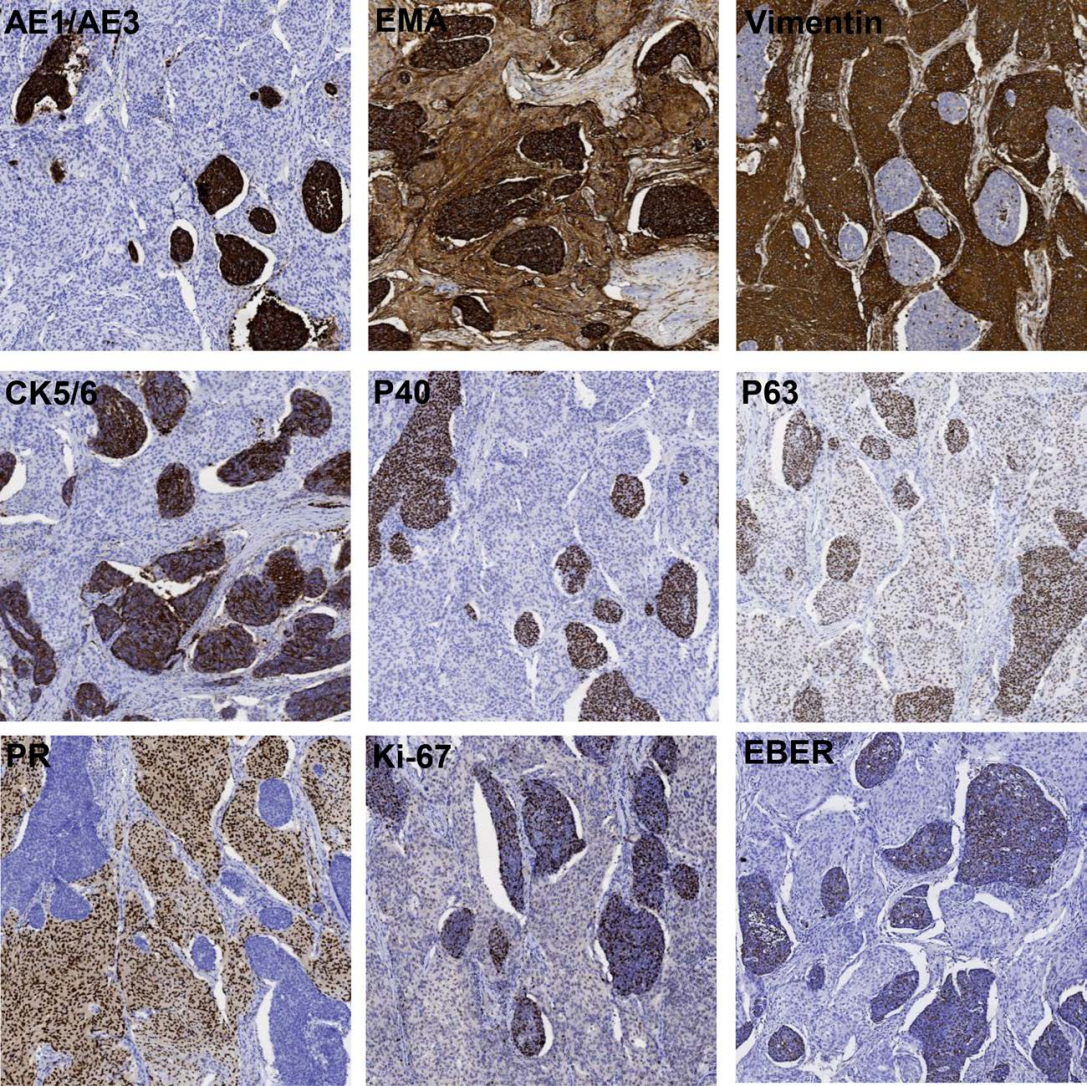
AE1/AE3, EMA, Vimentin, CK5/6, P40, P63, PR, Ki-67 expression and EBER expression of 2 distinct morphologies in the mass were showed by immunohistochemistry and ISH. AE1/AE3 = co-expressed cytokeratin, CK5/6 = cytokeratin 5/6, EBER = Epstein-Barr virus encoded RNA, EMA = epithelial membrane antigen, ISH = in situ hybridization, PR =progesterone receptor.

Histopathological examination revealed two distinct different morphologies. The first component (Fig. 2A) featured spindle cells arranged in parallel, fascicular and staggered. The cells were uniform in size, with delicate chromatin, rare mitoses and meningeal-like structure in local area. The interstitial was abundant with small blood vessels. The second component (Fig. 2B) featured epithelioid cells in the form of nests and sheets. The cells were distinct atypia with large size, unclear borders, round or oval nuclei, prominent nucleoli and increased mitotic activity. Pathological mitoses in these cells and interstitial lymphocytic infiltration were easily observed. There was no obvious transition between the 2 components and epithelioid cells could be observed in spindle cells (Fig. 2C). As shown in Figure 3, immunohistochemistry of spindle cells was positive for Vimentin, epithelial membrane antigen, progesterone receptor, which confirming the diagnosis of meningioma. Immunohistochemistry of epithelioid cells was positive for co-expressed cytokeratin, epithelial membrane antigen, cytokeratin 5/6, P40 and P63, and in situ hybridization showed epithelioid cells were diffusely positive reacted on Epstein–Barr virus (EBV) encoded RNA, which confirming the association with EBV. Ki-67 expression of spindle cells and epithelial cells were 5% and 70%, respectively. The pathological diagnosis was metastasis of nasopharyngeal carcinoma (differentiated non-keratinizing squamous cell carcinoma) to meningioma, combining clinical information, morphology, immunohistochemistry and in situ hybridization.

## 3. Discussion

TTM, referring to metastasis from one tumor to another tumor, is a well-known but rare phenomenon in oncology.^[1]^ Since the first case of metastasis from bronchogenic carcinoma to meningioma was reported in 1930,^[4]^ the theory of TTM has been arisen. Although TTM is most likely to be confused with collision tumor and the 2 are frequently mixed in many literatures, Campbell et al^[1]^ defined the criteria of TTM which was commonly accepted: exclusion of lymphatic metastasis to lymphoma, histological evidence of 2 distinct tumors, and surround of 1 tumor by at least a small margin of the other tumor. In this case, morphology and immunohistology confirm the diagnosis of meningioma and nasopharyngeal carcinoma, respectively, and epithelioid cell nests of nasopharyngeal carcinoma were surrounded by spindle cells of meningioma in some area, which meets the criteria of TTM.

There is one primary tumor as the recipient tumor and another tumor as the donor tumor in TTM. Donor tumors are usually malignant because of their high aggressiveness, while recipient tumors can be benign or malignant. The most common malignant recipient tumor is renal cell carcinoma,^[5]^ while the most common benign recipient tumor is meningioma^[3]^ according to the previous reports. Meningioma, a tumor originating from meningothelial cells, accounts for 36% of the primary intracranial tumor and is mostly graded as World Health Organization grade I.^[2]^ There were hypotheses related to the reason why meningioma is the most common recipient tumor in TTM^[6–8]^: The lack of a host immune response within meningioma makes the tumor an immune haven for metastasis. The rich vascular supply, low metabolic rate and indolent growth, and high collagen and lipid content of meningioma provides a suitable soil for the growth of malignant tumor. In addition, it may be related to the interaction between signal transduction, hormones and enzymes. Tumors metastasis to meningioma have been reported like breast cancer,^[9,10]^ lung cancer,^[7,11]^ esophageal carcinoma,^[12]^ renal cell carcinoma,^[13]^ prostatic adenocarcinoma,^[14]^ thyroid carcinoma,^[15]^ angiosarcoma,^[16]^ and malignant melanoma.^[17]^ And breast cancer and lung cancer are the most common entities metastasizing to meningioma.^[18]^ To our knowledge, we describe the first case of nasopharyngeal carcinoma metastasizing to meningioma. Nasopharyngeal carcinoma, a malignant tumor originating from nasopharyngeal mucosa and closely related to EBV infection, includes non-keratinizing squamous cell carcinoma, keratinizing squamous cell carcinoma and basaloid squamous cell carcinoma. Clinically, patients with nasopharyngeal carcinoma often present with cervical lymphadenopathy as the first symptom, same as the patient in this case, because it often metastasizes to cervical lymph nodes. Distant metastasis commonly happens in bone, lung and liver.^[19]^ Nasopharyngeal carcinoma can also invade the cerebral through the skull base directly. However, intracranial metastasis is rare.^[20]^ In this case, there is no evidence that nasopharyngeal carcinoma directly invades the meningioma through the skull base regardless of anatomy, imaging or intraoperative findings, which was consistent with the diagnosis of metastasis of nasopharyngeal carcinoma to meningioma.

There are only a few reports on tumor-to-meningioma metastasis, and treatment standard has not been established. Surgical resection seems to be the first choice, followed by postoperative radiotherapy and chemotherapy sometimes. According to those reports, the prognosis of patients with tumor-to-meningioma metastasis is poor. In this case, the patient is stable and received chemotherapy and immunotherapy using toripalimab, capecitabine and cetuximab after the surgery for 3 months.

Tumor metastasis happens through lymphatic or blood vessels usually. The presence of blood-brain barrier and the lack of a lymphatic drainage system makes it more difficult for tumors metastasis to brain compared with metastasis to other organs.^[21,22]^ And tumor metastasis to intracranial tumor, such as meningioma, is rare. Although the 2 different components are well differentiated morphologically, and immunohistochemistry is also helpful, sometimes there may be a trap in selecting tissue for sectioning to observe. Moreover, radiology cannot find the presence of tumor within meningioma currently.^[10,18]^ Therefore, both clinicians and pathologists should be aware of the possibility of tumor-to-meningioma metastasis regardless of whether the patient has a history of malignancy.

## Author contributions

**Investigation:** Yanbo Dai, Xiaoming Xing.

**Project administration:** Xue Li.

**Resources:** Ming Jin.

**Supervision:** Ming Jin.

**Writing – original draft:** Xue Li.

**Writing – review & editing:** Xue Li.
